# Efficacy and Safety of Durvalumab plus Tremelimumab for Unresectable Hepatocellular Carcinoma: A Real-World Multicenter Observational Study

**DOI:** 10.32604/or.2026.083206

**Published:** 2026-08-13

**Authors:** Krittiya Korphaisarn, Kosin Wirasorn, Suebpong Tanasanvimon, Kijjakom Thanasombunsukh, Kunlatida Maneenil, Jirawat Thanestada, Nattaya Teeyapun, Jarin Chindaprasirt, Chirawadee Sathitruangsak, Teerada Siripoon, Phannin Tiraswasdichai, Chanchai Charonpongsuntorn, Passakorn Wanchaijiraboon, Wannisa Laosuangkoon, Patrapim Sunpaweravong, Ekapop Sirachainan, Charuwan Akewanlop

**Affiliations:** 1 Division of Medical Oncology, Department of Medicine, Faculty of Medicine Siriraj Hospital, Mahidol University, Bangkok, Thailand; 2 Division of Medical Oncology, Department of Internal Medicine, Faculty of Medicine, Khon Kaen University, Khon Kaen, Thailand; 3 Division of Medical Oncology, Department of Medicine, Faculty of Medicine Chulalongkorn University, Bangkok, Thailand; 4 Department of Medicine, Lampang Hospital, Lampang, Thailand; 5 Oncology Unit, Rajavithi Hospital, Faculty of Medicine of Rangsit University, Bangkok, Thailand; 6 Maharat Nakhon Ratchasima Hospital, Nakhon Ratchasima, Thailand; 7 Division of Medical Oncology, Department of Internal Medicine, Faculty of Medicine, Prince of Songkla University, Songkhla, Thailand; 8 Division of Medical Oncology, Department of Medicine, Faculty of Medicine Ramathibodi Hospital, Mahidol University, Bangkok, Thailand; 9 Sakon Nakhon Hospital, Sakon Nakhon, Thailand; 10 Division of Medical Oncology, Department of Internal Medicine, Faculty of Medicine, Srinakharinwirot University, Ongkharak, Nakhon Nayok, Thailand; 11 Phrapokklao Cancer Center of Excellence, Phrapokklao Clinical Research Center, Phrapokklao Genomic Laboratories, Phrapokklao Hospital, Mueang District, Chanthaburi, Thailand; 12 Udon Thani Hospital, Mueang Udon Thani, Thailand

**Keywords:** Hepatocellular carcinoma, durvalumab, tremelimumab, STRIDE regimen, real-world outcomes, immunotherapy

## Abstract

**Introduction:** Durvalumab plus tremelimumab (Durva/Treme) improved survival in the HIMALAYA trial for unresectable hepatocellular carcinoma (HCC), but real-world evidence remains limited. This study aimed to evaluate clinical outcomes of Durva/Treme in routine practice. **Methods:** This retrospective multicenter study included patients with unresectable or advanced HCC who received Durva/Treme through the Expanded Access Program in Thailand between August 2023 and November 2025. Treatment outcomes and adverse events (AEs) were analyzed and descriptively compared with the HIMALAYA trial. **Results:** Fifty patients were included; median age was 62 years and 80% were male. Etiologies included hepatitis B (44%), hepatitis C (30%), and nonviral liver disease (26%). Most patients had Child–Pugh A (92%), while 24% had macrovascular invasion, including five with Vp4 portal vein involvement. The objective response rate (ORR) and disease control rate (DCR) were 12% and 60%, respectively. Among 43 patients meeting HIMALAYA eligibility criteria, ORR and DCR were 12% and 56%, respectively. With a median follow-up of 28.9 months, median progression-free survival (mPFS) and overall survival (mOS) were 4.6 and 10.5 months in the overall cohort, and 6.9 and 14.0 months in the HIMALAYA-eligible subgroup. Any-grade AEs occurred in 76% of patients, with grade ≥ 3 AEs in 24%. Hepatitis was the most common toxicity (48% overall; 14% grade ≥ 3). **Conclusions:** Durva/Treme demonstrated clinically meaningful activity with manageable toxicity in unresectable HCC. Outcomes among HIMALAYA-eligible patients were broadly consistent with the pivotal trial, supporting the feasibility of this regimen in routine clinical practice.

## 1 Introduction

Hepatocellular carcinoma (HCC) is the sixth most common cancer and the third leading cause of cancer-related death globally, with approximately 830,000 deaths annually [1,2]. The disease burden is especially high in the Asia-Pacific region, including Thailand, where chronic hepatitis B virus (HBV) infection remains the predominant etiological factor. Hepatitis C virus (HCV) infection and non-viral causes such as non-alcoholic fatty liver disease (NAFLD) also contribute significantly to the incidence of HCC [3,4,5]. Most patients present with unresectable or advanced-stage disease, often complicated by underlying cirrhosis and impaired liver function, limiting therapeutic options and resulting in poor prognosis [1,2].

Historically, systemic therapies for advanced HCC were limited, sorafenib (SOR) being the standard first-line treatment for more than a decade [6,7]. Lenvatinib (LEN) later emerged as an alternative first-line option, demonstrating non-inferior overall survival (OS) to SOR with a distinct safety profile [8]. The advent of immune checkpoint inhibitors (ICIs) has since transformed the treatment landscape. The HIMALAYA trial, a phase III randomized study, demonstrated that Durvalumab plus Tremelimumab (Durva/Treme), a dual immune checkpoint blockade targeting PD-L1 and CTLA-4, respectively, provided superior OS compared to SOR, with a median OS (mOS) of 16.4 months versus 13.8 months. The combination showed an objective response rate (ORR) of approximately 20%, and a manageable safety profile [9]. Based on these results, Durva/Treme has been established as a first-line treatment option for patients with unresectable HCC.

However, clinical trial populations are highly selected and often differ from real-world patients in routine clinical practice. In particular, patients encountered in daily practice may present with poorer liver function, higher tumor burden, macrovascular invasion, or portal vein tumor thrombosis (PVTT), which are commonly observed in Southeast Asia but were underrepresented in pivotal trials. In addition, treatment outcomes in patients with Child–Pugh B liver function or advanced PVTT remain insufficiently characterized [10,11]. Therefore, real-world evidence is essential to better understand the effectiveness and safety of Durva/Treme in broader, unselected patient populations. This is particularly important in regions with a high prevalence of viral hepatitis–related HCC and more advanced disease at presentation.

In this multicenter retrospective study, we aimed to evaluate the clinical outcomes and safety profile of Durva/Treme in Thai patients with unresectable or advanced HCC treated under an Expanded Access Program (EAP). We also explored outcomes in clinically relevant subgroups, including patients with high-risk features, and compared descriptive results with those reported in the HIMALAYA trial to better contextualize real-world performance.

## 2 Materials and Methods

### 2.1 Study Design and Patient Population

This retrospective multicenter study included patients with unresectable or advanced HCC treated with Durva/Treme through the EAP in Thailand between August 2023 and November 2025. The study was approved by the respective ethics review boards of all participating institutions, and the requirement of informed consent was waived owing to the retrospective study design. Patient data were anonymized in accordance with the Declaration of Helsinki and applicable local regulations.

Ethics approval was obtained from Siriraj Hospital (COA No. 959/2025), Khon Kaen University (HE681701), Songkhla Hospital (REC No. 68-511-14-1), Chulalongkorn University (COA No. 0223/2026), Ramathibodi Hospital (MURA No. 2025/865), Phrapokklao Hospital (COA No. 034/69), Lampang Hospital (B011/2569), Rajavithi Hospital (COA No. 010/2569), Maharaj Nakhon Ratchasima Hospital (COA No. 029/2026), Sakon Nakhon Hospital (COA/No. 052/2568), Somdech Phra Debaratana Medical Center (SWUEC-691025), and Udon Thani Hospital (UDH REC No. 8/2569).

Eligible patients were adults (≥18 years) with radiologically or histologically confirmed unresectable or metastatic HCC who received at least one dose of Durva/Treme through the EAP. Patients with different liver function statuses (Child–Pugh A or B) and varying tumor burdens, including macrovascular invasion and PVTT, were included to reflect real-world clinical practice. Patients were excluded if they had incomplete clinical data, did not receive treatment after enrollment, or had insufficient follow-up for treatment response or safety assessment.

### 2.2 Treatment Regimen

Patients received Durva/Treme according to the dosing schedule defined in the HIMALAYA trial protocol [9]: a single priming dose of Treme 300 mg combined with Durva 1500 mg on day 1, followed by Durva 1500 mg every 4 weeks. Treatment continued until disease progression, unacceptable toxicity, or treatment discontinuation.

### 2.3 Data Collection

Clinical data were retrospectively collected from medical records, including demographics, liver disease etiology, Child–Pugh score, Eastern Cooperative Oncology Group (ECOG) performance status, tumor characteristics (including macrovascular invasion and portal vein involvement), and subsequent therapies after Durva/Treme. Subsequent systemic therapies were recorded descriptively and were not included as covariates in the survival analyzes. Treatment outcomes, including objective response rate (ORR), disease control rate (DCR), progression-free survival (PFS), and OS, were assessed. Radiologic response was evaluated according to the Response Evaluation Criteria in Solid Tumors (RECIST) version 1.1 [12] as defined by the European Organization for Research and Treatment of Cancer (EORTC, Brussels, Belgium). Adverse events (AEs) were graded according to the Common Terminology Criteria for Adverse Events (CTCAE) version 5.0 (National Cancer Institute, Bethesda, MD, USA) [13]. PFS was defined as the time from initiation of Durva/Treme therapy to radiologic disease progression or death from any cause, whichever occurred first. OS was defined as the time from treatment initiation to death from any cause. Patients without events were censored at the date of last follow-up.

PVTT was classified radiologically according to the Liver Cancer Study Group of Japan criteria [14] as Vp1–Vp4: Vp1, tumor thrombus distal to second-order portal branches; Vp2, involvement of second-order branches; Vp3, involvement of first-order branches (right or left portal vein); and Vp4, involvement of the main portal trunk and/or contralateral portal vein branch.

### 2.4 Baseline Inflammatory Markers (Exploratory Analysis)

Exploratory analyzes of inflammation-related biomarkers were performed to evaluate the association between baseline neutrophil-to-lymphocyte ratio (NLR) and platelet-to-lymphocyte ratio (PLR) with treatment outcomes. NLR and PLR were calculated from complete blood count parameters obtained within 14 days before initiation of Durva/Treme. Patients receiving systemic corticosteroids at baseline or with clinically significant inflammatory, infectious, or decompensated liver conditions were excluded when data were available. Patients were stratified using predefined cutoff values of 5 for NLR and 150 for PLR based on previously published studies in HCC and immunotherapy settings [15,16]. These cutoff values were selected to facilitate comparison with prior literature. Cohort-specific optimization analyzes were not performed because of the limited sample size.

### 2.5 HIMALAYA in-Criteria Subgroup (Exploratory Analysis)

For exploratory analyzes, patients were categorized according to eligibility criteria adapted from the HIMALAYA trial and applied in the EAP. The HIMALAYA trial eligibility framework was used as the reference standard [9]; however, subgroup classification in this retrospective study was based on clinically available variables, including Child–Pugh class and Vp4 PVTT.

Patients were classified as HIMALAYA in-criteria if they had Child–Pugh class A liver function and absence of Vp4 PVTT. Patients with Child–Pugh class B liver function and/or Vp4 PVTT were classified as HIMALAYA out-of-criteria, consistent with key exclusion criteria of the trial.

These analyzes were exploratory and descriptive in nature, and no adjustment for baseline confounders was performed.

### 2.6 Endpoints

The primary endpoints were ORR, DCR, PFS, and OS. ORR was defined as the proportion of patients who achieved complete or partial response. DCR was defined as the proportion of patients achieving complete response, partial response, or stable disease. PFS was defined as the time from the initiation of Durva/Treme therapy to radiologic disease progression or death from any cause, whichever occurred first. OS was defined as the time from treatment initiation to death from any cause. Patients without documented progression or death at the time of analysis were censored at the date of last radiologic assessment (for PFS) or last known follow-up (for OS). Secondary endpoints included safety and tolerability, assessed by incidence, severity, and treatment discontinuation due to AEs.

### 2.7 Statistical Analysis

Descriptive statistics were used to summarize patient demographics, clinical characteristics, and safety data. Survival outcomes, including PFS and OS, were estimated using the Kaplan–Meier method with the corresponding 95% confidence intervals (CIs). Subgroup analyzes were conducted based on PVTT status, underlying cause of cirrhosis, and clinical response. Comparisons with data from the HIMALAYA trial were performed descriptively. All statistical analyzes were conducted using SPSS version 29.0 (IBM Corp., Armonk, NY, USA). A two-sided *p*-value of <0.05 was considered statistically significant.

## 3 Results

### 3.1 Patient Characteristics

A total of 50 patients were included in the EAP program. The median age was 62 years, and 80% were male. The underlying etiologies of liver disease were hepatitis B (44%), hepatitis C (30%), and non-viral causes (26%). Most patients (92%) had preserved liver function (Child-Pugh A), while 8% were classified as Child-Pugh B (score 7). Macrovascular invasion was present in 24% of patients, with 5 cases exhibiting tumor thrombus in the main portal vein (Vp4). Baseline characteristics are summarized in Table 1.

**Table 1:** Baseline characteristic (STRIDE_EAP vs. STRIDE_HIMALAYA).

Category	STRIDE_EAP (N = 50)	STRIDE_HIMALAYA (N = 393)
Median age (range), year	62 (33–87)	65 (22–86)
Gender, n (%)		
Male	40 (80%)	327 (83.0%)
Female	10 (20%)	66 (17.0%)
ECOG PS, n (%)		
0	20 (40.0%)	244 (62.0%)
1	28 (56.0%)	148 (37.7%)
2	2 (4.0%)	1 (0.3%)
Etiology, n (%)		
Hepatitis B	22 (44.0%)	122 (31.0%)
Hepatitis C	15 (30.0%)	110 (28.0%)
Non-viral hepatitis	13 (26.0%)	161 (41.0%)
Child-Pugh score, n (%)		
A	46 (92.0%)	387 (98.5%)
B (7)	4 (8.0%)	4 (1.0%)
Other	-	2 (0.5%)
BCLC stage, n (%)		
B	10 (20.0%)	77 (20.0%)
C	40 (80.0%)	316 (80.0%)
Macrovascular invasion, n (%)		
Yes	12 (24.0%)	103 (26.2%)
Vp4	5 (10.0%)	-
Vp3	3 (6.0%)	-
Vp2	4 (8.0%)	-
No	38 (76.0%)	290 (73.8)
AFP > 400 ng/mL, n (%)	22 (44.0%)	145 (37.0%)

Abbreviations: AFP, alpha-fetoprotein; BCLC, Barcelona Clinic Liver Cancer; ECOG PS, Eastern Cooperative Oncology Group performance status; STRIDE, Single Tremelimumab Regular Interval Durvalumab; EAP, expanded access program; VP, portal vein tumor thrombosis classification.

### 3.2 Clinical Response

**Figure 1:**
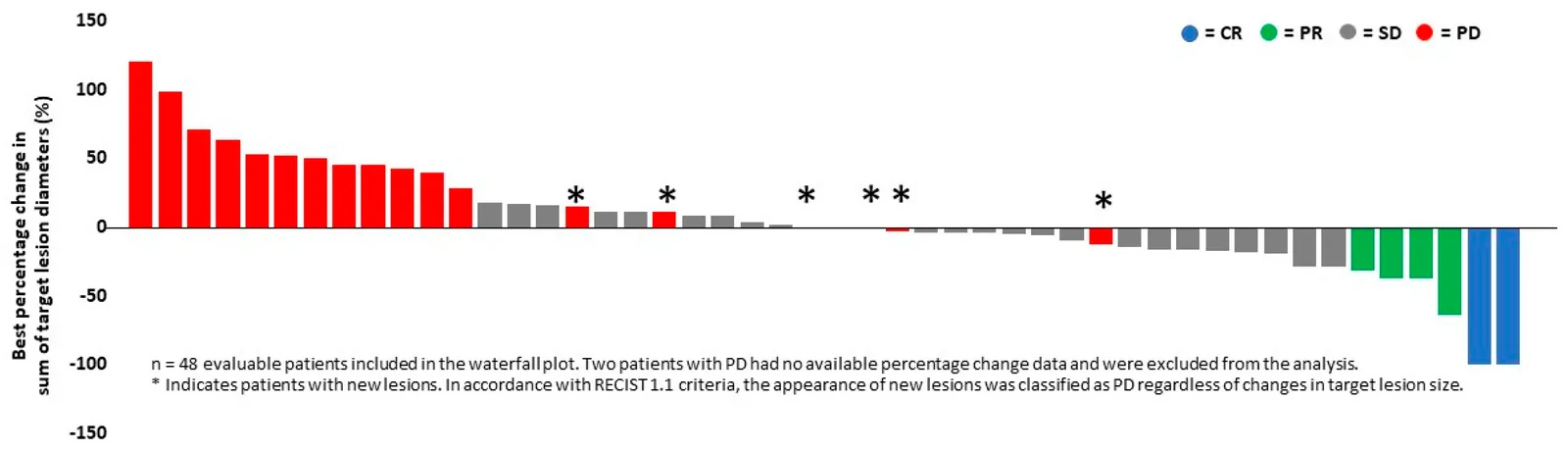
**Waterfall plot of the best percentage change in the size of the target lesion.** Each bar represents one patient and is ordered according to the maximum percentage change in tumor size. Tumor shrinkage was observed in a subset of patients. Most patients demonstrated either disease stabilization or tumor shrinkage, whereas a smaller proportion showed disease progression, indicating heterogeneous treatment response to Durva/Treme in this cohort. *indicates patients with new lesions. In accordance with RECIST 1.1 criteria, the appearance of new lesions was classified as PD regardless of changes in target lesion size.

The overall ORR was 12% (95% CI, 4–24%), including 2 patients (4%) with CR and 4 patients (8%) with PR. The DCR was 60% (95% CI, 45–73%). Among 43 patients who met HIMALAYA eligibility criteria, the ORR was 12% and DCR was 56%. Two patients without available radiologic tumor measurements were excluded from the waterfall plot analysis. In accordance with RECIST 1.1 criteria, the appearance of new lesions was classified as PD, regardless of changes in target lesion size. Detailed response outcomes are shown in Table 2, and the waterfall plot is shown in Fig. 1.

**Table 2:** Efficacy Outcomes Comparison (EAP vs. HIMALAYA).

Parameter	EAP (N = 50)	HIMALAYA (N = 393)	HIMALAYA In-Criteria (N = 43)
**Median follow-up (months)**	28.9 (95% CI, 28.3–29.5)	33.2	—
**Response by RECIST 1.1, n (%)**			
** CR**	2 (4.0%)	12 (3.1%)	—
** PR**	4 (8.0%)	67 (17.0%)	—
** SD**	24 (48.0%)	157 (39.9%)	—
** PD**	20 (40.0%)	157 (39.9%)	—
**ORR, % (95% CI)**	12.0 (4–24)	20.1	12.0
**DCR, % (95% CI)**	60.0 (45–73)	60.1	56.0
**mPFS months (95% CI)**	4.6 (2.1–7.1)	3.8 (3.7–5.3)	6.9 (2.9–10.9)
**mOS months (95% CI)**	10.5 (6.8–14.3)	16.4 (14.2–19.6)	14.0 (7.9–20.1)

Abbreviations: CI, confidence interval; CR, complete response; DCR, disease control rate; EAP, expanded access program; mOS, median overall survival; mPFS, median progression-free survival; ORR, objective response rate; PD, progressive disease; PR, partial response; RECIST 1.1, Response Evaluation Criteria in Solid Tumors version 1.1; SD, stable disease.

### 3.3 Patient Disposition and Subsequent Therapy

The most common reason for discontinuation was progressive disease (PD), accounting for 42 patients (84%). AEs led to discontinuation in 4 patients (8%), while death from causes other than the study condition was recorded in 1 patient (2%). At the time of analysis, 3 patients (6%) were still ongoing in the study. Following disease progression, 21 patients received subsequent systemic therapy. Lenvatinib was the most commonly used agent (n = 9), followed by FOLFOX chemotherapy (n = 8). Additionally, 1 patient received cabozantinib, and 3 patients were treated with sorafenib. Details of treatment discontinuation and subsequent therapy are presented in Table 3.

**Table 3:** Treatment Discontinuation and Subsequent Therapy.

Category	Subcategory	N (%)
**Discontinuation reason**	Progressive disease	42 (84%)
	Adverse events	4 (8%)
	Death from other cause	1 (2%)
	Ongoing	3 (6%)
**Subsequent therapy**	No	29 (58%)
	Yes	21 (42%)
	Lenvatinib	9 (18%)
	Cabozantinib	1 (2%)
	Sorafenib	3 (6%)
	FOLFOX	8 (16%)

Abbreviations: FOLFOX, folinic acid, fluorouracil, and oxaliplatin.

### 3.4 Survival Outcomes

**Figure 2:**
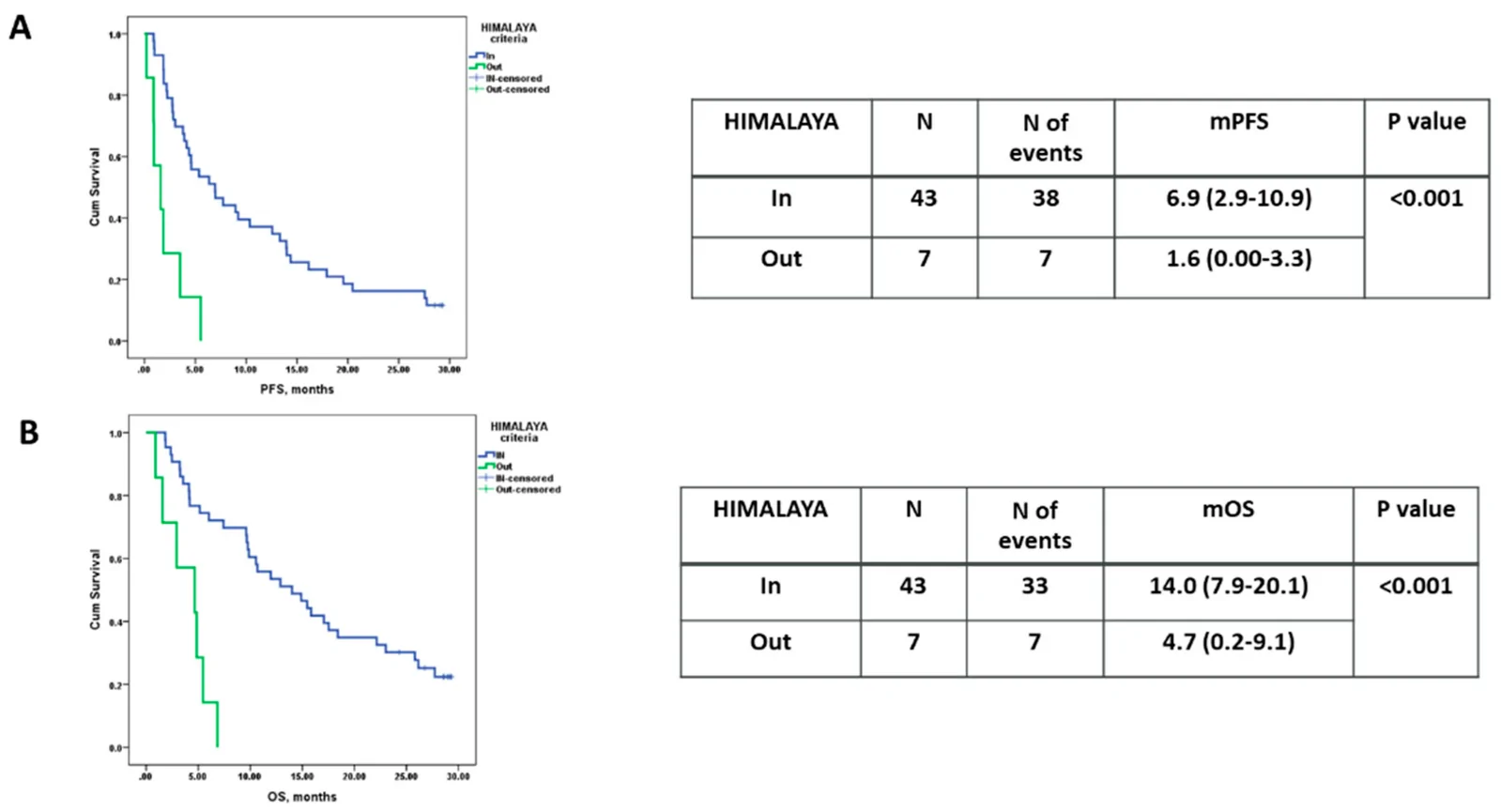
**Kaplan–Meier survival analysis by HIMALAYA eligibility criteria.** Kaplan–Meier curves of progression-free survival (mPFS) (**A**) and overall survival (mOS) (**B**) stratified by HIMALAYA in-criteria and out-of-criteria groups.

At a median follow-up of 28.9 months (95% CI, 28.3–29.5), 10 patients (20%) were still alive. In the overall cohort, median PFS (mPFS) was 4.6 months (95% CI, 2.1–7.1), and mOS was 10.5 months (95% CI, 6.8–14.3). In the HIMALAYA in-criteria subgroup, mPFS was 6.9 months (95% CI, 2.9–10.9), and mOS was 14.0 months (95% CI, 7.9–20.1). These outcomes were descriptively consistent with, although numerically lower than, those reported in the HIMALAYA trial. Efficacy outcomes are summarized in Table 2 and Fig. 2.

### 3.5 Subgroup Analyzes

**Figure 3:**
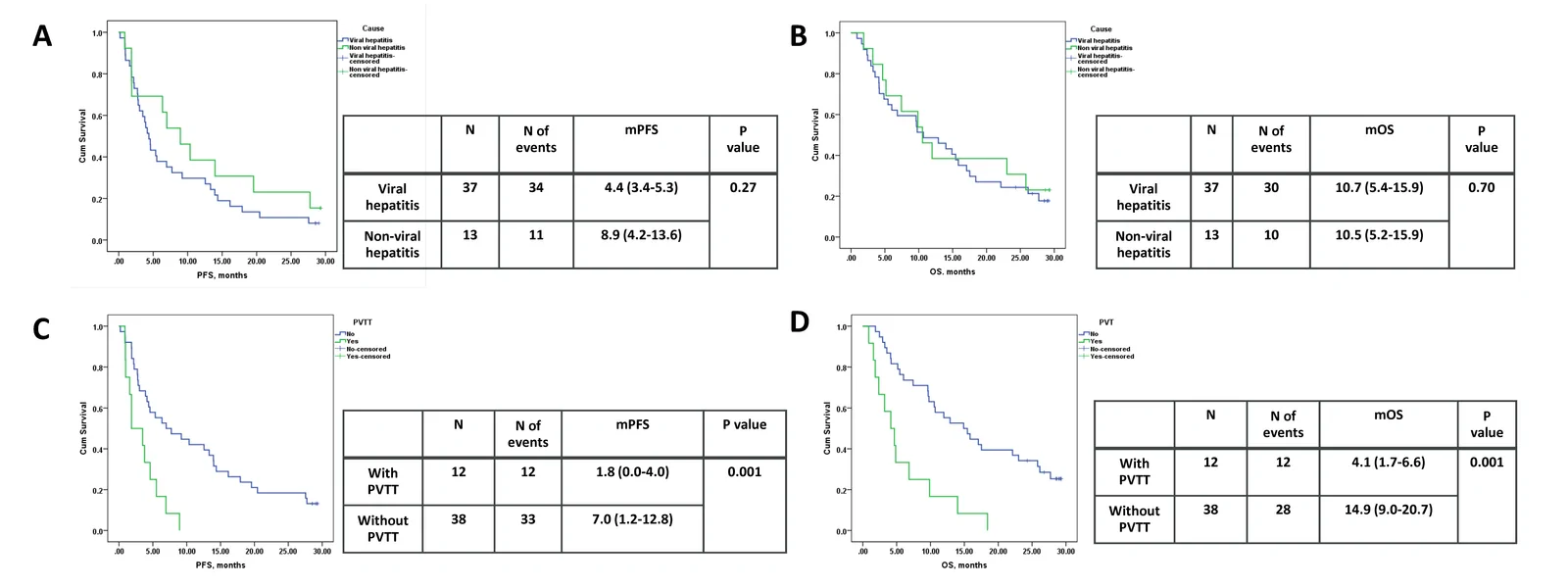
**Kaplan–Meier survival analysis by clinical subgroup.** Kaplan–Meier curves of progression-free survival (PFS) and overall survival (OS) stratified by underlying etiology of cirrhosis and portal vein thrombosis (PVTT) status. (**A**) mPFS and (**B**) mOS according to underlying etiology of cirrhosis. (**C**) mPFS and (**D**) mOS according to PVTT status.

Patients with nonviral etiologies exhibited numerically longer mPFS compared with those with viral hepatitis (8.9 vs. 4.4 months; *p* = 0.27), although no clear difference in OS was observed. In contrast, the presence of PVTT was strongly associated with poorer outcomes. Patients with PVTT had shorter mPFS (1.8 vs. 7.0 months; *p* = 0.001) and mOS (4.1 vs. 14.9 months; *p* = 0.001) compared with those without PVTT (Fig. 3).

### 3.6 Association between Response and Survival

**Figure 4:**
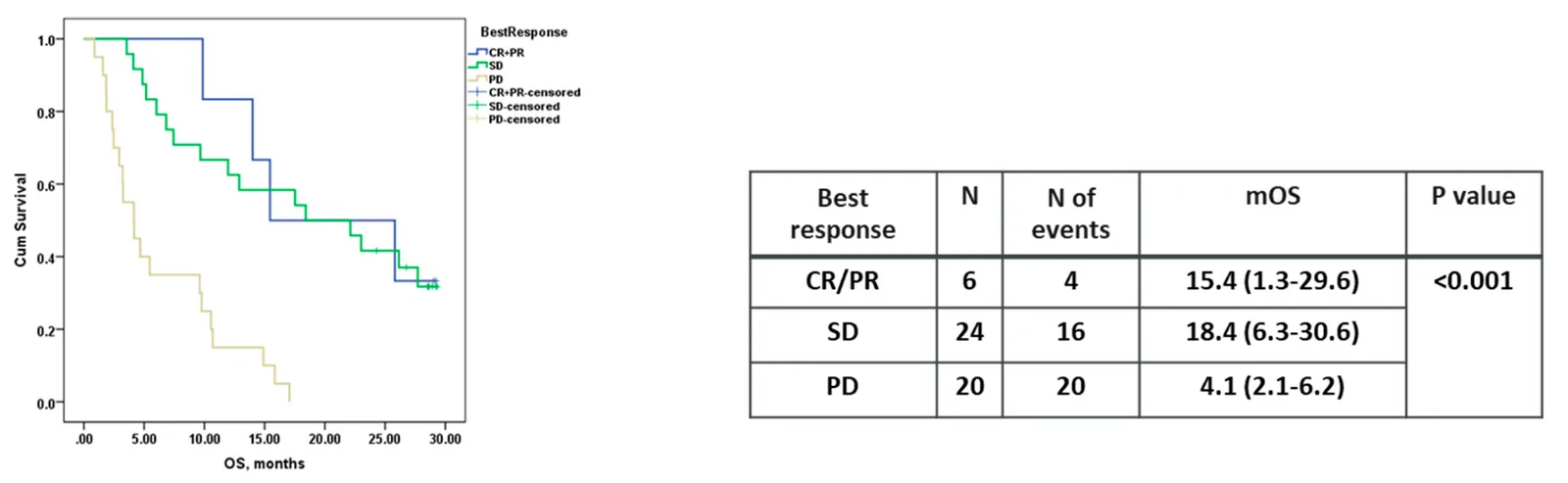
**Kaplan–Meier analysis of overall survival according to the best overall response.** Kaplan–Meier curves of overall survival (OS) stratified by best overall response. Patients with progressive disease (PD) demonstrated the shortest survival. Patients who achieve complete response (CR), partial response (PR), or stable disease (SD) showed relatively better and overlapping survival outcomes, indicating variability in overall survival across response categories in patients treated with Durva/Treme.

Survival outcomes differed according to best overall response (*p* = 0.001) (Fig. 4). The mOS was 15.4 months (95% CI, 1.3–29.6) in patients achieving complete or partial response, 18.4 months (95% CI, 6.3–30.6) in those with stable disease, and 4.1 months (95% CI, 2.1–6.2) in those with progressive disease.

### 3.7 Association of Baseline NLR and PLR with Survival

No statistically significant associations were observed between baseline NLR or PLR and survival outcomes. Patients with NLR ≥ 5 had a mPFS of 5.5 months and mOS of 12.9 months, compared with 4.1 months and 10.5 months, respectively, in patients with NLR < 5 (PFS: *p* = 0.51; OS: *p* = 0.98). Similarly, patients with PLR ≥ 150 had a mPFS and mOS of 5.4 and 10.5 months, respectively, compared with 4.1 and 9.9 months in patients with PLR < 150 (PFS: *p* = 0.92; OS: *p* = 0.95) (Appendix A Table A1).

### 3.8 Adverse Events

In the overall cohort, 76% of patients (n = 38) experienced at least one AE of any grade, while 24% (n = 12) experienced grade ≥ 3 AEs. The most common laboratory abnormalities were elevations in aspartate aminotransferase (AST) and alanine aminotransferase (ALT), observed in 48% (n = 24) and 44% (n = 22) of patients, respectively. Grade ≥ 3 elevations occurred in 12% and 14% of patients. Diarrhea was reported in 6% (n = 3) of patients, with one case (2%) classified as grade ≥ 3. Endocrine immune-related AEs, including hypothyroidism and hyperthyroidism, were each observed in 14% of patients, all of which were grade 1–2. Immune-related AEs were less common but notable for their severity. Myocarditis, myositis, adrenal insufficiency, and hypophysitis were each reported in 1–2 patients (2–4%), and all were grade ≥ 3. No cases of pneumonitis were observed. Treatment-related AEs are summarized in Table 4.

**Table 4:** Adverse Events.

Category	EAP (N = 50)		HIMALAYA (N = 393)	
Adverse Events, n (%)	Any Grade	Grade ≥ 3	Any Grade	Grade ≥ 3
Any AE	38 (76.0)	12 (24.0)	378 (97.4)	196 (50.5)
Anemia	10 (20.0)	3 (6.0)	36 (9.3)	11 (2.8)
Diarrhea	3 (6.0)	1 (2.0)	103 (26.5)	17 (4.4)
Rash	12 (24.0)	0 (0.0)	87 (22.4)	6 (1.5)
AST elevation	24 (48.0)	6 (12.0)	48 (12.4)	20 (5.2)
ALT elevation	22 (44.0)	7 (14.0)	36 (9.3)	10 (2.6)
Increase bilirubin	8 (16.0)	4 (8.0)	20 (5.2)	3 (0.8)
Hypothyroidism	7 (14.0)	0 (0.0)	47 (12.1)	0 (0.0)
Hyperthyroidism	7 (14.0)	0 (0.0)	18 (4.6)	1 (0.3)
Myocarditis	1 (2.0)	1 (2.0)	-	2 (0.5)
Myositis	1 (2.0)	1 (2.0)	-	3 (0.8)
Pneumonitis	0 (0.0)	0 (0.0)	5 (1.3)	4 (1.0)
Adrenal insufficiency	2 (4.0)	2 (4.0)	6 (1.5)	1 (0.3)
Hypophysitis	1 (2.0)	1 (2.0)	-	-

Abbreviations: AE, adverse event; ALT, alanine aminotransferase; AST, aspartate aminotransferase; EAP, expanded access program.

## 4 Discussion

In this multicenter real-world cohort, Durva/Treme demonstrated clinically meaningful activity with a manageable safety profile in patients with unresectable HCC. Survival outcomes in patients meeting the HIMALAYA in-criteria were broadly consistent with those reported in the pivotal trial, providing supportive real-world evidence for the applicability of the HIMALAYA regimen in routine clinical practice.

ICI–based combinations are now established as first-line therapy for advanced HCC. In the HIMALAYA trial, Durva/Treme achieved a mOS of 16.4 months, with an ORR of 20.1% and a DCR of 60.1% [9]. In our cohort, mOS (10.5 months) and ORR (12%) were lower, whereas DCR (60%) was comparable. These differences should be interpreted cautiously, as our study included a broader, more heterogeneous population than the pivotal trial.

A key factor likely contributing to these differences is baseline patient selection. Our cohort included patients with more advanced disease features, including macrovascular invasion (24%) and Vp4 PVTT (10%), which were not represented in the HIMALAYA trial. In addition, 8% of patients had Child–Pugh B liver function, whereas only Child–Pugh A patients were included in the trial. These differences reflect routine clinical practice in Asia, where patients often present with more advanced disease and compromised hepatic reserve.

Importantly, when analysis was restricted to patients who met HIMALAYA eligibility criteria, the survival outcomes improved, with mPFS of 6.9 months and mOS of 14.0 months. These results are broadly consistent with, although still numerically lower than, those reported in the trial, supporting the external validity of Durva/Treme in appropriately selected patients. However, given the retrospective design and differences in follow-up and monitoring intensity, cross-trial comparisons should be interpreted cautiously.

Subgroup analyzes demonstrated heterogeneity in treatment outcomes. Patients with nonviral etiologies showed a numerically longer mPFS compared with those with viral hepatitis, although this did not translate into an OS difference. This finding is consistent with the HIMALAYA trial [9] and suggests that dual immune checkpoint blockade retains activity across etiologic subtypes. Differences in tumor immune microenvironment between viral and non-viral HCC, particularly NAFLD-related disease, may partly explain variable responses and warrant further investigation [17,18,19].

In contrast, the presence of PVTT was strongly associated with a poor prognosis. Patients with PVTT had markedly reduced mPFS (1.8 months) and mOS (4.1 months) compared with those without PVTT. This represents one of the most clinically significant findings of our study and reinforces PVTT—especially advanced Vp4 disease—as a major determinant of poor outcome in HCC. Although Durva/Treme remained feasible in this population, the observed clinical benefit appeared limited, underscoring the need for more effective treatment approaches for patients with extensive vascular invasion. Prospective studies are warranted to better define optimal therapeutic strategies for this high-risk subgroup.

Survival outcomes also varied according to treatment response. Patients achieving disease control (CR, PR, or SD) had significantly longer survival compared with those with progressive disease. Interestingly, the longest survival was observed in patients with stable disease, which may reflect durable immunologic disease control and tumor biology heterogeneity. Similar patterns have been observed in other immunotherapy studies, where stable disease can still translate into meaningful long-term survival benefit [9,20,21,22,23]. Conversely, patients with primary progressive disease had poor outcomes, with mOS of approximately 4 months, emphasizing the importance of early response assessment and the need for predictive biomarkers. In exploratory analyzes, baseline inflammatory markers (NLR and PLR) were not significantly associated with survival outcomes. Although no statistically significant differences were observed, the analysis was limited by the small sample size and should be interpreted as hypothesis-generating.

The safety profile observed in this study was generally consistent with that of the HIMALAYA trial, although the incidence of AEs was lower in our cohort. This likely reflects differences in retrospective data collection, real-world monitoring intensity, and potential underreporting of low-grade toxicities. Hepatic enzyme elevations were relatively more frequent, which may be attributable to underlying cirrhosis and reduced hepatic reserve in this population. Immune-related AEs were uncommon but clinically significant when present, including rare grade ≥ 3 endocrinopathies and cardiac toxicities. Overall, treatment discontinuation due to AEs remained low (8%), supporting the manageable safety profile of Durva/Treme in routine clinical practice.

Our findings are consistent with emerging real-world evidence demonstrating similar efficacy and safety of Durva/Treme across diverse populations. A Japanese cohort of 44 previously untreated patients with advanced HCC reported comparable ORR (15.8%) and DCR (53.3%) [24]. Similarly, the multicenter DT-Real study [25] confirmed reproducible efficacy and safety in 233 patients, 80% of whom received first-line therapy, reporting an ORR of 23.7% and a DCR of 64.9%, with improved survival among patients meeting HIMALAYA in-criteria (median OS: 23.0 vs. 12.2 months; median PFS: 6.6 vs. 3.9 months). More recently, real-world data from the US Veterans Administration STRIDE cohort further support the effectiveness of Durva/Treme in routine clinical practice, demonstrating clinically meaningful overall survival in an unselected population, including patients with heterogeneous liver function and comorbidities, thereby reinforcing its external validity beyond clinical trial settings [26]. Although these studies consistently support the clinical utility of Durva/Treme, important differences in patient selection, baseline liver function, and tumor burden limit direct cross-study comparisons. Collectively, these findings strengthen the external validity of the HIMALAYA trial and underscore the importance of careful patient selection when translating clinical trial evidence into real-world practice.

Beyond HIMALAYA, other immune-based combinations, including atezolizumab plus bevacizumab (Atezo/Bev) and nivolumab plus ipilimumab (Nivo/Ipi), have also demonstrated efficacy in first-line treatment of advanced HCC and provide important benchmarks for interpretation. The IMbrave150 trial reported a mOS of 19.2 months and ORR of 30% with Atezo/Bev [21]. However, the anti-angiogenic component is associated with an increased risk of bleeding, limiting its use in patients with untreated or high-risk esophageal varices. In contrast, the CheckMate 9DW trial reported an ORR of 36% and a mOS of 22.8 months with Nivo/Ipi in previously untreated patients [20], although this regimen was associated with a higher incidence of grade ≥ 3 immune-related AEs (53%), requiring careful patient selection and close monitoring.

In our prior real-world Thai cohort treated with Atezo/Bev [27], mOS and mPFS were 10.2 and 6.7 months, respectively, which were broadly comparable to the present outcomes observed with Durva/Treme, with similar DCR (Appendix A Table A2). These findings are consistent with a large multicenter real-world study of 452 patients [28], which demonstrated no significant difference in survival outcomes between Atezo/Bev and Durva/Treme in the first-line setting (mOS 14.0 vs. 14.6 months: *p* = 0.66), supporting comparable effectiveness across regimens in routine clinical practice. Nevertheless, such cross-trial and cross-regimen comparisons should be interpreted cautiously due to inherent differences in study populations, study design, and follow-up.

Given the modest ORR and survival outcomes observed in our cohort, strategies to improve the effectiveness of Durva/Treme in routine practice warrant further investigation. Combination with anti-angiogenic agents may enhance antitumor activity through synergistic modulation of the tumor microenvironment. In addition, integration with locoregional therapies such as transarterial chemoembolization, radiotherapy, or ablation may further improve treatment response through tumor antigen release and immune priming effects, particularly in patients with high tumor burden or PVTT [29,30,31]. Prospective studies are needed to clarify the optimal role of these combination approaches in advanced HCC.

Importantly, treatment selection in clinical practice should extend beyond efficacy alone and incorporate patient-specific factors, including comorbidities and contraindications. For example, Atezo/Bev may be less suitable for patients with a high risk of bleeding or untreated esophageal varices, whereas Durva/Treme may be preferable in such settings due to the absence of anti-angiogenic therapy. Although Atezo/Bev and Nivo/Ipi may achieve higher response rates in selected populations, their distinct toxicity profiles and eligibility requirements limit universal applicability. Therefore, Durva/Treme remains an important first-line option with a balanced efficacy–safety profile and broader usability in routine practice, particularly in patients with portal hypertension or contraindications to anti-angiogenic therapy. In the absence of head-to-head randomized trials, treatment decisions should be individualized based on tumor burden, liver function, bleeding risk, comorbidities, and healthcare resource considerations.

Overall, our study supports the external validity of the HIMALAYA regimen in real-world practice while also highlighting important differences in patient characteristics and outcomes compared with clinical trials. These findings emphasize the importance of appropriate patient selection and the need for prospective studies to further refine optimal treatment strategies, particularly in high-risk subgroups such as patients with PVTT or impaired hepatic reserve.

## 5 Limitations

This study has several limitations. Its retrospective design and relatively small sample size may introduce selection bias and limit statistical power. The absence of a comparator arm means that all comparisons with the HIMALAYA trial and other studies are descriptive and should be interpreted cautiously. In addition, potential underreporting of low-grade AEs cannot be excluded in this real-world setting. Variability in imaging intervals, follow-up schedules, and the lack of central radiologic review may also have influenced response assessment. Furthermore, heterogeneity in subsequent therapies after disease progression may have affected survival outcomes. Subgroup analyzes, including those evaluating inflammation-based biomarkers such as NLR and PLR, were exploratory and limited by small sample sizes, and should therefore be interpreted with caution. Despite these limitations, the study provides meaningful real-world evidence on the use of Durva/Treme in advanced HCC.

## 6 Conclusion

Durva/Treme demonstrated clinically meaningful activity and manageable toxicity in a real-world Thai cohort of advanced HCC. Clinical outcomes appeared modestly inferior to those reported in randomized trials, potentially reflecting broader patient eligibility and a higher-risk disease population encountered in routine practice. Nevertheless, the regimen may represent a reasonable first-line option, particularly for patients who are unsuitable for anti-angiogenic therapy. These findings provide additional real-world data supporting the use of the HIMALAYA regimen in clinical practice.

## Data Availability

The data that support the findings of this study are available from the Corresponding Author, Krittiya Korphaisarn, upon reasonable request.

## Abbreviations

AEs	Adverse Events
Atezo/Bev	Atezolizumab Plus Bevacizumab
CTCAE	Common Terminology Criteria for Adverse Events
DCR	Disease Control Rate
Durva/Treme	Durvalumab Plus Tremelimumab
ECOG	Eastern Cooperative Oncology Group
EAP	Expanded Access Program
HBV	Hepatitis B Virus
HCV	Hepatitis C Virus
HCC	Hepatocellular Carcinoma
ICIs	Immune Checkpoint Inhibitors
LEN	Lenvatinib
mPFS	Median PFS
mOS	Median OS
NLR	Neutrophil-to-Lymphocyte Ratio
Nivo/Ipi	Nivolumab Plus Ipilimumab
NAFLD	Non-Alcoholic Fatty Liver Disease
ORR	Objective Response Rate
OS	Overall Survival
PLR	Platelet-to-Lymphocyte Ratio
PVTT	Portal Vein Tumor Thrombosis
PD	Progressive Disease
PFS	Progression-Free Survival
RECIST	Response Evaluation Criteria in Solid Tumors
SOR	Sorafenib

## Appendix A

**Table A1:** Association of baseline NLR and PLR with survival.

Variable	mPFS (months)	95% CI	*p* Value	mOS (months)	95% CI	*p* Value
**NLR ≥ 5**	5.5	3.0–8.0	0.51	12.9	2.2–20.6	0.98
**NLR < 5**	4.1	0.6–7.6		10.5	7.7–13.4	
**PLR ≥ 150**	5.4	3.3–7.4	0.92	10.5	5.7–15.4	0.95
**PLR < 150**	4.1	0.0–8.8		9.9	0.0–18.9	

Abbreviations: CI, confidence interval; mPFS, median progression-free survival; mOS, median overall survival; NLR, neutrophil-to-lymphocyte ratio; PLR, platelet-to-lymphocyte ratio; mos, months.

**Table A2:** Efficacy and safety for Durva/Treme and Atezo/Bev in the Thai EAP program.

Category	Durva/Treme (N = 50)		Atezo/Bev (N = 30)	
**Median follow-up time (mo) (95% CI)**	**28.9 (95% CI, 28.3–29.5)**		**10.1 (95% CI, 6.9 to NA)**	
**Response by RECIST1.1, n (%)**				
** CR**	2 (4.0)		0 (0.0)	
** PR**	4 (8.0)		7 (23.3)	
** SD**	24 (48.0)		12 (40.0)	
** PD**	20 (40.0)		11 (36.7)	
**ORR (%)**	12.0		23.3	
**DCR (%)**	60.0		63.3	
**mPFS, mo**	4.6		6.7	
**mOS, mo**	10.5		10.2	
**Reason for discontinuation, n (%)**				
** PD**	42 (84.0)		22 (73.3)	
** SE**	4 (8.0)		4 (13.3)	
** Death from another cause**	1 (2.0)		3 (10.0)	
** Other**	0 (0.0)		1 (3.0)	
** Ongoing**	3 (6.0)		-	
**Adverse events of interest, n (%)**	**Any Grade**	**Grade ≥ 3**	**Any Grade**	**Grade ≥ 3**
**Any AE**	38 (76.0)	12 (24.0)	-	-
** Anemia**	10 (20.0)	3 (6.0)	8 (26.7)	-
** Diarrhea**	3 (6.0)	1 (2.0)	5 (16.7)	-
** Rash**	12 (24.0)	0 (0.0)	3 (10.0)	-
** Hypertension**	-	-	12 (40.0)	1 (3.3)
** Proteinuria**	-	-	10 (33.3)	2 (6.7)
** AST or ALT elevation**	24 (48.0)	7 (14.0)	6 (20.0)	1 (3.3)
** Hypothyroidism**	7 (14.0)	0 (0.0)	5 (16.7)	-
** Gastrointestinal hemorrhage**	-	-	6 (20.0)	-
** Myocarditis**	1 (2.0)	1 (2.0)	-	-
** Polymyositis**	1 (2.0)	1 (2.0)	-	-

Abbreviations: NA, not applicable; AE, adverse event; ALT, alanine aminotransferase; AST, aspartate aminotransferase; Atezo/Bev, atezolizumab plus bevacizumab; CR, complete response; DCR, disease control rate; Durva/Treme, durvalumab plus tremelimumab; EAP, expanded access program; mOS, median overall survival; mPFS, median progression-free survival; ORR, objective response rate; PD, progressive disease; PR, partial response; RECIST 1.1, Response Evaluation Criteria in Solid Tumors version 1.1; SD, stable disease.
